# A Case Report of Hyperestrogenism in Prader-Willi Syndrome

**DOI:** 10.1016/j.aace.2021.06.003

**Published:** 2021-06-12

**Authors:** Isabella Albanese, Natasha Garfield

**Affiliations:** 1 Department of Medicine, McGill University, Montreal, Quebec, Canada; 2 Department of Medicine, McGill University, Division of Endocrinology, McGill University Health Center, Montreal, Quebec, Canada

**Keywords:** Prader-Willi syndrome, PCOS, estrogen, testosterone, FSH, follicle-stimulating hormone, HRT, hormone replacement therapy, LH, luteinizing hormone, PCOS, polycystic ovary syndrome, PWS, Prader-Willi syndrome

## Abstract

**Objective:**

Prader-Willi syndrome (PWS) is associated with multiple endocrinopathies, including hypogonadism. The mechanism underlying hypogonadism in PWS is thought to be secondary to hypothalamic dysfunction, primary gonadal defect, or a combination of both. Here, we present a case of hyperestrogenism in PWS due to concomitant polycystic ovary syndrome (PCOS) and therapeutic considerations regarding hormone replacement therapy (HRT).

**Case Report:**

An 18-year-old woman with PWS transferred to adult care from pediatrics was found to have hyperestrogenism (specifically, elevated estrone with normal estradiol levels). Additionally, she demonstrated oligomenorrhea and hyperandrogenism, meeting diagnostic criteria for PCOS. After 3 months of therapy with cyclic medroxyprogesterone alone, she developed normal withdrawal bleeding.

**Discussion:**

Given the elevated estrone and normal estradiol levels, our patient’s hyperestrogenism is thought to be a direct result of her hyperandrogenism due to peripheral conversion. Prolonged exposure to unopposed estrogen is an established risk factor for endometrial cancer development in PCOS; thus, this was taken into account regarding her HRT, and she was treated with cyclic progesterone alone.

**Conclusion:**

Women with PWS are typically treated with combined estrogen and progesterone HRT; however, our case, a unique presentation of PCOS in PWS, demonstrated the importance of tailoring HRT to a patient’s specific needs.

## Introduction

Prader-Willi syndrome (PWS) is a rare genetic disorder caused by the absence of paternal gene expression at the chromosomal region 15q11.2-q13, with concomitant epigenetic inactivation of the same maternal genes. This is associated with a range of clinical manifestations such as hypotonia, dysmorphic features, developmental delay, disordered behavior, hyperphagia, progressive obesity, and obstructive sleep apnea. PWS is associated with multiple endocrinopathies, including growth hormone deficiency, metabolic syndrome, type 2 diabetes mellitus, central hypothyroidism, central adrenal insufficiency, osteoporosis, and hypogonadism.^1^

Hypogonadism is a hallmark of PWS, manifesting clinically as genital hypoplasia, delayed or incomplete puberty, and infertility. Hypogonadism in PWS is thought to be secondary to a variable combination of hypothalamic dysfunction and primary gonadal defect.^2^ However, there have been rare cases of spontaneous gestation in women with PWS, demonstrating that fertility is possible in these individuals.^3^ Despite this, low to low-normal estrogen and luteinizing hormone (LH) levels are consistently reported in pubertal women with PWS. Follicle-stimulating hormone (FSH) levels are variable depending on the patient’s mechanism of hypogonadism. Androgen levels have been noted to be slightly elevated in childhood but generally normalize in adulthood.^4^

## Case Report

An 18-year-old woman with PWS, confirmed by genetic testing, was transferred to adult care from the pediatric hospital. She demonstrated multiple clinical features of PWS such as hyperphagia, short stature, learning disabilities, behavioral issues, and episodes of self-mutilation. Past medical history was otherwise only significant for obstructive sleep apnea and obesity (body mass index, 49 kg/m^2^). She had menarche at the age of 13 years with subsequent secondary amenorrhea (periods of >9 months without menstruating). Her pertinent investigations have been summarized in the Table. Because of her high estrogen and estrone levels, she was started on cyclic medroxyprogesterone treatment every 3 months, which resulted in normal withdrawal bleeding on this therapy. She also uses topical clindamycin/benzoyl peroxide for acne and had never been treated with growth hormone therapy. She also cited a gradual history of hirsutism over her trunk without virilization.

**Table:** Pertinent Investigations

Hormone	Value	Normal range
Estradiol, pmol/L	381	Midfollicular: 99-448 Periovulatory: 349-1590 Midluteal: 180-1068 Postmenopausal: <147
Estrone, pmol/L	649	Follicular: 111-370 Luteal: 180-420 Menopausal: <148
Total testosterone, nmol/L	3.37	Women aged >20 years: <0.7-3.5
Dehydroepiandrosterone sulfate, μmol/L	10.8	Women aged 18-50 years: 2-8.1
17-Hydroxyprogesterone, nmol/L	3.3	Follicular: 1-2 Luteal: 2-7 Menopausal: 0.4-1.5
Luteinizing hormone, IU/L	<0.2	Follicular: 1.2-11.6 Midcycle: 17.5-49.6 Luteal: 0.6-10 Menopausal: 29.7-72.4
Follicle-stimulating hormone, IU/L	<0.2	Follicular: 1.6-9.2 Midcycle: 4.8-28.6 Luteal: 0.8-3.6 Menopausal: 28-99.7
Prolactin, μg/L	9.5	3.3-26.7
Thyroid-stimulating hormone, mIU/L	2.12	0.4-4.4
Free thyroxine, pmol/L	10.3	8-18
Insulin-like growth factor 1, nmol/L	29.5	13.3-42.6
Random cortisol, nmol/L	135	12-535

## Discussion

In PWS, hypogonadotropic hypogonadism and primary ovarian insufficiency are the commonly cited causes of hypogonadism and amenorrhea. Our patient’s FSH and LH levels were noted to be low; however, she demonstrated both hyperestrogenism and hyperandrogenism. This pattern is neither consistent with hypogonadotropic hypogonadism, in which low FSH, LH, and estrogen levels would be expected, nor primary ovarian insufficiency, in which elevated FSH and LH levels with low estrogen level would be the expected pattern. Regarding her hyperestrogenism, although estradiol levels were within the normal range, estrone level was elevated. Estrone is an estrogen that predominantly comes from peripheral conversion of androgens, whereas estradiol production is mostly ovarian. The 17-hydroxyprogesterone level was normal, thus eliminating nonclassical 21-hydroxylase deficiency as the cause of her hyperandrogenism. Given our patient’s oligomenorrhea and biochemically confirmed hyperandrogenism, she meets the Rotterdam diagnostic criteria for polycystic ovary syndrome (PCOS). To our knowledge, there have been no other reported cases of PCOS in PWS. An important consideration in the management of PCOS is that prolonged exposure to unopposed estrogen increases the risk of endometrial cancer.^5^ Despite the fact that amenorrhea in PWS is typically treated with combined estrogen and progesterone replacement, taking our patient’s hyperestrogenism into account, she was instead treated with cyclic medroxyprogesterone alone, resulting in normal withdrawal bleeding.

## Conclusion

Despite her PWS diagnosis, our patient demonstrated hyperestrogenism, as opposed to hypoestrogenism, which characterizes PWS. This is likely as a result of peripheral conversion of testosterone secondary to concomitant PCOS. This is clinically relevant regarding her hormone replacement therapy. Amenorrhea in PWS is typically treated with combined estrogen and progesterone replacement; however, our patient only requires cyclic progesterone. This case demonstrated that, in PWS, oligomenorrhea can have various etiologies and health care providers can consider tailoring hormone replacement therapy to a patient’s specific needs.

## Disclosure

The authors have no multiplicity of interest to disclose.
